# Improving Newborn Survival in Low-Income Countries: Community-Based Approaches and Lessons from South Asia

**DOI:** 10.1371/journal.pmed.1000246

**Published:** 2010-04-06

**Authors:** Nirmala Nair, Prasanta Tripathy, Audrey Prost, Anthony Costello, David Osrin

**Affiliations:** 1 Ekjut, Chakradharpur, West Singhbhum, Jharkhand, India; 2 UCL Centre for International Health and Development, Institute of Child Health, University College London, United Kingdom

## Abstract

David Osrin and colleagues discuss the critical importance of reducing global neonatal mortality in developing countries and how community-based approaches can help.

**Summary Points**

- Reducing global neonatal mortality is crucial. In low-income countries, most births and deaths occur at home.
- Obstacles to improving survival include: many newborn infants are invisible to health services; care-seeking for maternal and newborn ailments is limited; health workers are often not skilled and confident in caring for newborn infants; and there are inequalities across all these factors.
- The best community-based approach is a combination of community mobilization and home visits by community-based workers. Both timing of visits and treatment interventions are critical.
- It is not clear how community-based approaches should be balanced, and whether they are effective outside South Asia and when introduced into public sector systems. Operational challenges include integrating community-based activities into public health systems, and questions of how to achieve coverage at scale.
- The possibility of partnership between the public and nongovernment sectors should be explored, particularly in terms of novel large-scale collaborations.

## The Scale of the Problem

Until about twenty years ago, child survival meant the survival of children rather than newborn infants. With a steady worldwide decline in under-5 deaths—most of the lives saved being those of infants and children over the age of a month—the newborn period has come into focus as a relatively intransigent source of mortality. The “child survival revolution” increased child survival [1], but newborn infants went largely unnoticed. Neonatal mortality (0–28 d) now accounts for about two-thirds of global infant (0–1 y) mortality and about 3.8 million of the 8.8 million annual deaths of children under 5 [2]. Most of these deaths (98%) occur in low- and middle-income countries [3].

The last two decades have seen a rise in advocacy—a call for attention to the newborn infant along with her mother and siblings—and an incremental growth in the evidence for potential interventions [4]–[6]. Reducing neonatal mortality is both an ethical obligation and a prerequisite to achieving Millennium Development Goal 4, the target of which is a reduction in child mortality by two-thirds between 1990 and 2015. A 2008 report found only a quarter of relevant countries on track to reach this target [7].

## Immediate Challenges

The main obstacles to improving newborn survival are that many babies are born at home without skilled attendance, care-seeking for maternal and newborn ailments is limited, health workers are often not skilled and confident in caring for newborn infants, and inequalities in all these factors are felt by those most in need.

### Home Births and Limited Access to Care

In low-income settings, most babies are born at home and more than half of those who die do so at home. Three-quarters of neonatal deaths occur in the first week, and just under half in the first 24 h [3]. In South Asia and East and Southern Africa, only about 35% of births take place in institutions [8]. The newborn infant has traditionally occupied a transitional space between potential and actual personhood, and seclusion practices add to the likelihood that he or she will be invisible to health professionals. If care is sought, it is often in the traditional sector and beset by obstacles such as the notion that mother and baby are polluted, which may entail seclusion and cause delay in care-seeking. Access to allopathic (“Western” or biomedical) health services is limited by lack of facilities, human resources, equipment, and consumables.

There are four general ways of addressing this: improving the provision and quality of institutional health care, extending institutional care through community outreach, stimulating demand for appropriate health care and institutional delivery through community engagement and perhaps financial incentives, and changing ideas and behaviour by working with communities. These approaches are far from mutually exclusive and should be joined up [9].

### Content of Health Care

The three commonest causes of neonatal deaths are infections (28%), complications of prematurity (30%), and intrapartum-related (“birth asphyxia”) (24%) [2]. Unfortunately, health workers may lack the skills and experience necessary to act appropriately. Basic resuscitation skills and knowledge may be limited, and there is a pervasive idea that intervention needs to be highly technical. This is not generally true. As early as 1905, Budin recommended resuscitation, warmth, early and frequent breastfeeding, keeping the baby with his or her mother, hygiene, and prompt recognition and treatment of illness [10]. Contemporary recommendations for “essential newborn care” follow this blueprint [11],[12]. *The Lancet*'s series on neonatal survival suggested that between 41% and 72% of neonatal deaths could be averted if 16 simple, cost-effective interventions were delivered with universal coverage. Among these are adequate nutrition, improved hygiene, antenatal care, skilled birth attendance, emergency obstetric and newborn care, and postnatal visits for mothers and infants [13].

### Inequity

Newborn survival increases with wealth. In India, for example, neonatal mortality is 56 per 1,000 in the poorest quintile, but 25 in the richest [14]. Such inequality is evident no matter how the population is segmented: by education, ethnicity, migrant status, or occupation [15]. In many countries, the responsibility to provide health care for poorer people falls on the public sector. As wealthier members of society move steadily towards private sector care, the burden of care for increasing numbers of poor people falls on already overstretched national public health systems.

## What Is Meant by Community Intervention?

**Figure 1:**
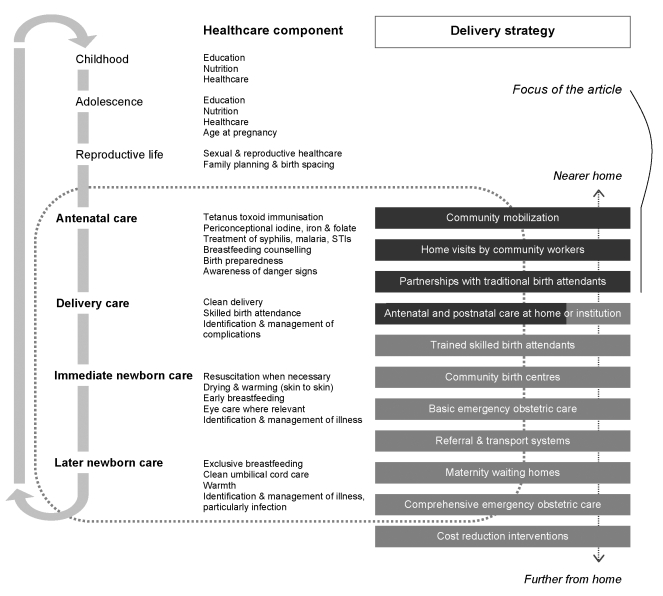
Maternity as a life event, components of care with potential effects on newborn survival, and 11 possible delivery strategies.

Figure 1 summarises both the components of health care that have been recommended to improve newborn survival and the range of delivery strategies that have been proposed, tested, or introduced. More detail can be found in a recent set of systematic reviews on intrapartum-related deaths [16]–[20]. Pregnancy is just one stage of a woman's life, and the figure reminds us that it may occur on a background of gender inequality. Inadequate education, nutrition, and care for childhood illness have short- and long-term effects that are not limited to women (although the burdens often fall on them, as do young age at marriage and conception, short birth intervals, and undesirably large families).

The figure locates intervention strategies in terms of their proximity to a woman's home. Some approaches deserve fuller comment than we can give here. Antenatal care is, and should remain, a feature of health care systems. It allows contact between women and health workers and strengthens the likelihood of birth preparedness and institutional delivery. It may identify certain remediable issues, and although its effectiveness in terms of general risk reduction is debated [21],[22], it can be delivered in the home. The same is true of postnatal care, and both phases of ambulatory care are included in community-based interventions. Training skilled birth attendants is central to current efforts to improve maternal outcomes and is included in health plans in many low-income countries. However, the rate of output is limited and unlikely to answer demand in the next 10 y [23]. Linked with this is the provision of skilled intrapartum care at primary health care centres, which is the focus of recommendations for maternal survival [24]. Both transport and referral remain problematic in many countries.

The issue is not only geographical movement between health care institutions but also realisation that a problem exists and communication and decision-making for referral. A maternity waiting home is a residence near a hospital to which women at risk move shortly before delivery or if complications arise [25]. The benefits have not been demonstrated conclusively, risk screening may be of limited use, and community acceptance varies, but waiting homes are an option in some settings and a strategy adopted in Cuba, for example. Cost reduction is an overarching means of stimulating demand for health services [26]. Strategies include the removal of user fees [27], conditional cash transfers for use of services [28], and insurance schemes.

All the potential approaches serve communities, but we will focus on the beginning of the sequence close to home (highlighted in Figure 1), in which the essential feature is not primarily institutional. We do so in response to a number of recent research programs—most of them successful and all of them originating in low-income countries—and the incorporation of their findings into national and international guidelines. It is true that community-driven approaches fall somewhat outside the prevailing health sector paradigm (in reality if not in principle), but they raise important questions about integration, which we will discuss later. Table 1 summarises published controlled trials in which the interventions under test included one or more of three broad strategies: community mobilization initiatives, programmes that involved home visits by community-based workers, and partnerships with traditional birth attendants (TBAs).

**Table 1:** Components of interventions and key features of controlled trials of community-based approaches to improve newborn survival.

Who Did the Intervention?	What Did They Do?	Population Involved	Evaluation Design	Neonatal Mortality Rate Effect: Odds Ratio (95% CI)
**Bangladesh: Beanibazar, Zakiganj and Kaighat subdistricts, Sylhet ** [**38**]		480,000	Cluster RCT	
*“Home care”*				0.7 (0.5–0.9)
Community health worker	Identified pregnancies through surveillance every 2 wk.			
	Made 2 antenatal home visits.			
	Provided iron and folic acid supplements.			
	Made 3 postnatal home visits.			
	Identified illness in infants.			
	Managed sepsis with injectable antimicrobials and referred.			
	Completed management if referral was unsuccessful.			
Female community mobiliser	Facilitated community group meetings every 4 mo.			
Male community mobiliser	Facilitated community group meetings.			
*“Community care”*				No effect seen
Female community mobiliser	Facilitated community group meetings every 8 mo.			
Male community mobiliser	Facilitated community group meetings every 10 mo.			
Community resource person	Identified pregnant women.			
	Encouraged meeting attendance.			
**India: Gadchiroli, Maharashtra ** [**40**],[**41**]		80,000	Single control	0.3 (0.2–0.4)
Community health worker	Identified pregnant women.			
	Conducted group health education.			
	Made 2 antenatal home visits.			
	Attended delivery, gave vitamin K injection.			
	Made 8–12 postnatal home visits.			
	Weighed infants and identified and managed high-risk infants (birth asphyxia, sepsis, low birth weight).			
	Encouraged appropriate referral.			
	Recorded monitoring information.			
Supervisor	Supervised community health workers every 15 d.			
**India: Shivgarh, Uttar Pradesh ** **[37]^a^**		104,000	Cluster RCT	0.5 (0.4–0.6)^b^
Community health worker	Facilitated initial community meetings.			
	Facilitated monthly community folk song meetings.			
	Facilitated monthly newborn care meetings with stakeholders and community volunteers.			
	Identified pregnancies 3-monthly door-to-door.			
	Made 2 antenatal home visits.			
	Made 2 postnatal home visits.			
	Advised on care seeking			
Supervisor	Supported community health workers.			
Community volunteer	Supported home visits and community meetings.			
Community role model	Supported home visits and community meetings.			
**India: Barabanki & Unnao districts, Uttar Pradesh ** [**61**]		45,000	Single control	No effect
Auxiliary nurse midwife	Registered pregnancies.			
	Made 3 antenatal home visits.			
	Conducted deliveries.			
	Made postnatal home visit.			
Integrated Child Development Services worker	Recruited community volunteers.			
	Registered pregnancies.			
	Made 3 antenatal home visits.			
	Made postnatal home visit.			
	Gave food supplements.			
Community volunteer	Made 3 antenatal home visits.			
	Made postnatal home visit.			
Traditional birth attendant	Conducted deliveries.			
**Nepal: Makwanpur district ** [**32**]		170,000	Cluster RCT	0.7 (0.5–0.9)
Community facilitator	Activated and facilitated monthly community women's groups.			
Supervisor	Supported facilitators.			
**Pakistan: Larkana district, Sindh ** [**50**]		1,300,000	Cluster RCT	0.7 (0.6–0.8)
Traditional birth attendant	Made 3 antenatal home visits.			
	Registered pregnant women with lady health worker.			
	Used delivery kits.			
Lady health worker	Supported traditional birth attendant.			
	Enrolled and followed up pregnancies.			
	Recorded outcomes.			
Obstetrician	Trained traditional birth attendants.			
	Ran 8 outreach clinics in 6 mo.			
**Pakistan: Hala & Matiari subdistricts, Sindh ** [**39**]		139,000	Cluster RCT (pilot)	0.7 (0.6–0.9)^c^
Lady health worker	Conducted community group education.			
	Identified pregnancies.			
	Provided basic antenatal care.			
	Made 2 antenatal home visits.			
	Made 5 postnatal home visits.			
	Identified and managed danger signs.			
	Linked with traditional birth attendant.			
Traditional birth attendant	Gave basic newborn care.			
	Attended community group education.			
Community volunteer	Set up community health committees.			
	Emergency transport fund.			
	3 monthly community group education.			

a Intervention 2 added liquid crystal thermometry by community health workers.

b Rate ratio.

c Comparison was pre-post intervention, not intervention-control.

CI, confidence interval; RCT, randomised controlled trial.

### Community Mobilization

All the suggested approaches to improving newborn survival involve a degree of community mobilization. While general community development programmes may improve newborn survival, our experience in India and Nepal suggests that survival-focused interventions may reduce neonatal mortality rates even more efficiently. Here we emphasise programs in which work with communities to identify problems and solutions is a specific strategy to increase newborn survival. The idea that communities can develop insight into and solutions for their own problems has a long history and social and political implications [29],[30]. A stimulus for newborn survival initiatives came from Bolivia's Warmi program, which worked with rural Aymara women's groups to identify local opportunities and develop strategies to improve maternal and newborn health [31]. Groups moved through a cycle of discussions that encompassed sharing of experiences, internalising new information, prioritising, strategising, action, and evaluation. In a modified version of this process, in rural Nepal, a cluster randomised trial suggested that women's groups facilitated by a local female community worker—trained in facilitation techniques but without a health care background—could reduce neonatal mortality rates by about 30% [32]. There were behaviour changes in, for example, hygienic practices and care-seeking for problems, and also strategic initiatives such as maternal and child health funds and transport schemes. Seventy-five percent of groups remained active 18 mo after withdrawal of program support. The model is being tested with rural groups in Bangladesh [33], India [34], and Malawi [35] and in urban slums in India [36]. Group work, not necessarily confined to women, and with varying degrees of intensity, was also a feature of other successful programs [37],[38],[39].

### Home Visits by Community Workers

Aside from the benefits of group-based discursive approaches, a growing number of programs have shown that targeted home visits by community-based workers can help reduce newborn mortality. The idea developed over some years in rural Maharashtra, India, where the nongovernment organisation (NGO), the Society for Education, Action and Research in Community Health (SEARCH) trained community health workers to conduct group health education, identify pregnant women and make antenatal care visits to their homes, attend delivery, give vitamin K injections, make several further postnatal home visits, identify and manage infants at risk from birth asphyxia, low birth weight and sepsis, and encourage appropriate referral. This seminal model gradually reduced neonatal mortality by 70% [40],[41].

Like most successful local initiatives, the SEARCH approach developed incrementally in the context of a commitment to community development and included a range of activities. The most prominent were regular visits to women and their newborn infants by a cadre of community-based women trained and remunerated by SEARCH. These local nongovernment workers were able to give advice and identify and treat neonatal problems, their skills extending to resuscitation and administration of intramuscular antibiotics. Since then, trials of home-based care have been conducted in North India [37], Bangladesh [38], and Pakistan [39] (summarised along with other key work in Table 1). Strategies differed in personnel and content. All the programs included community meetings, antenatal and postnatal home visits, and preventive advice. The Hala program included referral [39], as did the Projahnmo program, which also included curative care [35]. Strategies were also implemented by different cadres of workers. The Shivgarh strategy involved community health workers remunerated by the program and local volunteers [37]; the Projahnmo strategy involved NGO community health workers and mobilisers [38]; and the Hala strategy involved government Lady Health Workers, TBAs, and community volunteers.

Most of the programs showed improvements in care: increased uptake of antenatal care, some increase in institutional delivery (although this was not a primary feature of any program), and better performance on indicators of essential newborn care. Further evaluations are underway in South and Southeast Asia (Bangladesh, India, Nepal, Pakistan, and Vietnam) and sub-Saharan Africa (Ethiopia, Ghana, Malawi, Mali, Mozambique, South Africa, Tanzania, and Uganda), and WHO and UNICEF now recommend home visits in the first week of life by appropriately trained and supervised health workers [42].

### Partnerships with Traditional Birth Attendants

About 60 million infants are delivered outside institutions annually, 23%–40% of them by TBAs [19], women who deliver babies in the community, with or without clinical training [43]. The idea of bringing TBAs into the allopathic fold by upgrading their skills and connecting them with health services has had a chequered history. Included in programs from the time of Alma Ata [44],[45], subsequent review led to the virtual abandonment of TBA training, or at least a modification of their role from care providers to link-workers [46],[47]. Recent reviews suggest that traditional attendants could have a role in increasing newborn survival [45],[48],[49], and a controlled trial in rural Pakistan found a 30% reduction in neonatal mortality when they were linked systematically with government community health workers and obstetric services [50]. There is also evidence that infants could be saved if TBAs had some skills in managing birth asphyxia, for example [51].

## Five Things That We Need to Know

### The Correct Balance of Supply and Demand Intervention

No attempt to address newborn deaths in the home will be successful if it does not reach the household and align with the aspirations of family members [52]–[54]. How much of the agenda should be community-driven, and how much should be predefined by health sector inputs, is still not clear. At one extreme, Nepal's Makwanpur trial worked through women's group discussions and the resulting interventions were left to community members to decide [32]. Maharashtra's SEARCH program involved a portfolio of interventions developed over several years (training of TBAs, home visits by community health workers, identification of illness, and administration of oral and parenteral antibiotics). Perceptions of the most important intervention differ according to the commentator. For some, the key issue was the provision of injectable antimicrobials by community-based workers (perhaps responsible for 30%–40% of the mortality reduction) [55]. For others, the essential transformation was due to the longevity of the program and the commitment of its cadres, driven by deeply held beliefs about community rights and action. Both must have played a part, and community group work has (rightly, we think) been included in all successful programs for newborn care. India's Shivgarh trial made community mobilization integral to the intervention, while in Bangladesh's Projahnmo trial, group activities were limited to visits made by female community mobilisers every 4 mo. The former trial showed an effect and the latter did not; integration and coverage seem to be important.

### What Is Needed Outside South Asia

All the major trials of community interventions for newborn survival have so far been in South Asia. Their commonalities are more striking than their differences: rural setting, female literacy at around 40%, home delivery rates over 80%, skilled birth attendance below 15%, and public sector health care systems based on the primary health care model. Africa needs more attention, not least because the pattern of mortality may be different. Low birth weight—a key contributor to neonatal mortality in South Asia—is much less common in African countries, and post-neonatal mortality claims a greater share of under-5 deaths [56]. We hope that the operational research and trials underway in African countries, mentioned above, will answer some of the questions about whether strategies are both feasible and effective on another continent.

### How to Fit Components into Systems

It is possible to think about community interventions in at least three ways: as a series of activities that need to be delivered (“package”), as a framework for delivery (“system”), or as a means of galvanising communities for change (“mobilization”). The 16 recommended newborn care practices are best seen as a package of activities, and there have been recent attempts to refine its content: antenatal care and birth preparedness, institutional delivery if possible, hygiene, early wiping and wrapping of the infant (but delayed bathing), early and exclusive breastfeeding, skin-to-skin contact between mother and infant, and recognition and appropriate treatment when danger signs appear [57]. What is required is integration of family, community, health system outreach and institutional care, and also of maternal, newborn, child, adolescent, and women's health care into a systemic continuum [8],[57]–[59]. Examining individual components is not the same as evaluating the effects of delivering them within complex systems. A recent review found no true effectiveness trials conducted at scale in health systems and few studies approximating complete packages; current evidence was “a weak foundation for guiding effective implementation of public health programmes addressing neonatal health,” and the reviewers called for new effectiveness trials tailored to local health needs and conducted at scale in developing countries [60].

### Whether Workers Can Cope with the Intensity Demanded

Programs have worked so far with institutional cadres, community-based workers, and volunteers. In some cases the community-based workers were a new cadre [32],[37],[38],[40],[41], while in others they were drawn from existing public sector cadres [39],[50],[61]. As Table 1 shows, programs have usually involved more than one of these groups. Once a precedent is set—often a portfolio of activities—the options for less intensive approaches become questionable. Several of the model programs required at least two home visits during pregnancy, a visit on the day of birth, and at least three postnatal home visits [38],[39],[40],[41]. It is not clear how much pruning models would stand and still remain effective. Given the importance of the first days after birth to neonatal mortality, perhaps dropping the later postnatal visits would not compromise the outcomes [37],[50],[61]. In a recent joint statement, WHO and UNICEF recommend a minimum of two visits, in the first 24 h and on the third day [42].

Most programs involve an increased workload for community cadres and a substantial contribution from volunteers. While existing community-based health workers may achieve more job satisfaction from clearly delineated activities and support, increased workloads may be challenging, particularly because of the requirement for extensive field activities. Haines and colleagues have described problems in instituting focused tasks, adequate remuneration, training, and supervision in large-scale community health-worker programs [62]. Less than 15% of children born at home in five South Asian and sub-Saharan African countries were visited by a trained health worker within 3 d of birth. Speed of reaction and mobility might also be obstacles: a community health worker, perhaps living in a different village, must know about a woman's pregnancy or be informed of the birth and must then be willing and able to make repeated postnatal visits to check for warning signs in mother and baby and to treat or refer promptly. It is hard to know if this will happen, particularly since these activities have been part of the augmented surveillance systems for some trials [37],[38]. Villages are heterogeneous, and vulnerable marginalized groups may be less likely to be visited at home when programs expand beyond trial models with more rigorous supervision.

### Coverage at Scale

Successful model programs need to be replicated, scaled up, and sustained. Although cost is usually a prime concern in this sort of discussion, it has not so far been a major obstacle. The interventions proposed are relatively inexpensive and could be integrated with existing systems [23],[58]. It is health systems integration that raises questions. Child survival is unequivocally important, and several countries have developed newborn care policies. Government partners are involved in operational research in Bangladesh, India, Indonesia, Nepal, Vietnam, Ethiopia, Malawi, Mali, Mozambique, Bolivia, and Guatemala (Saving Newborn Lives/Save the Children, personal communication). In Nepal, the NGO Mother and Infant Research Activities (MIRA) has embarked on a large trial in which NGO-employed facilitators of women's groups are replaced by existing female community health volunteers. SEARCH has supported two pilot scale-up programs, one mediated through NGOs at seven sites in Maharashtra (ANKUR) and one nested within the public health systems of five states (Indian Council of Medical Research field trial). The findings of the Shivgarh trial have been integrated into Uttar Pradesh's child survival program, and home-based newborn care has been included in both the Government of India's Reproductive and Child Health (RCH-II) strategy and the Integrated Management of Newborn and Child Illness.

Putting aside the issues of the content of programs and the continuum of care, the main challenge is to achieve the required levels of community mobilization and home visits by community-based workers. As with many public health interventions, it is the least accessible groups (geographically, socially, financially) who have the most problems and for whom outreach is most likely to be compromised if corners are cut. Only 13% of women who deliver at home in developing countries make a postnatal care visit [63]. The first priority is for community newborn survival interventions to be included in public sector health services. Here we face a tacit assumption that programs spearheaded by NGOs will not be viable or scalable; that the inertia of health systems will thwart efforts to build community linkages and generate enthusiasm and conscientiousness. Partnerships between the government and third (nongovernment) sectors could help. The success of NGOs in Bangladesh, for example, has been unprecedented in the country, with nationwide reach for organisations such as BRAC (www.brac.net) and private not-for-profit organisations such as the Diabetic Association of Bangladesh (www.dab-bd.org). Savings and credit initiatives have led to the creation and sustenance of thousands of community groups across Asia, many of them run by and for women. Cross-system linkage has been difficult, and one of the central agendas is to evaluate the possibilities of collaboration between sectors so that health care systems are integrated rather than parallel. Clearly, government needs to be involved in creating an enabling environment for such movements, perhaps only insofar as endorsement, but preferably through collaboration and policy. For example, India's National Rural Health Mission, which will work through community-based Accredited Social Health Activists (ASHAs), represents an opportunity for creative cross-sectoral partnership.

## The Next Stage

There is little doubt that community interventions for newborn survival work in principle. In our opinion, the key questions are now more about the medium than the message: how effective simplified program designs might be, whether they are relevant in African contexts, whether they will be as effective as they appear, and how they could be rolled out and sustained. Research now needs to move from components to the operational realities of systems [60],[64]. Some major questions remain: the optimal population coverage of community-based workers, since coverage, we think, is crucial for success, and does require investment in community mobilization [65],[66], the requirements for selection of workers and their remuneration or compensation [41], and the right mix of existing and new cadres [12]. A particular challenge is how to integrate newborn and maternal survival interventions [67]. For governments the choice of approach should almost certainly focus on defining the roles and responsibilities of existing cadres in reaching out to women who deliver at home with an essential newborn care package. This is not simply a matter of training health workers, since it is the marginalised and hard to reach who are most at risk. Women's groups represent a valuable community resource that already exists in many areas and may have inbuilt sustainability. We see active involvement of individuals and communities as the key to achieving targeted coverage of poor and marginalized families to bring down neonatal mortality, and this is an opportunity for governments to facilitate community mobilization in partnership with civil society organisations.

**Box. Five Things That We Need to Know**

1. The correct balance of supply and demand intervention
2. What is needed outside South Asia
3. How to fit components into systems
4. Whether workers can cope with the intensity demanded
5. Coverage at scale
